# Two decades (1998 to 2018) of collaborative human immunodeficiency virus clinical pharmacology capacity building in a resource constrained setting

**DOI:** 10.1186/s12962-021-00327-y

**Published:** 2021-11-10

**Authors:** Charles C. Maponga, Tsitsi G. Monera-Penduka, Takudzwa J. Mtisi, Robin Difrancesco, Faithful Makita-Chingombe, Fine Mazambara, Kathleen Tooley, Tinashe Mudzviti, Gene D. Morse

**Affiliations:** 1 International Pharmacotherapy Education and Research Initiative (IPERI), Department of Pharmacy and Pharmaceutical Science, University of Zimbabwe Faculty of Medicine and Health Sciences, c/o Drug and Toxicology Information Services (DaTIS), PO Box A178, Avondale Harare, Zimbabwe; 2 Center for Integrated Global Biomedical Sciences, School of Pharmacy and Pharmaceutical Sciences, Translational Pharmacology Research Core, New York State Center of Excellence in Bioinformatics and Life Sciences, The State University of New York, Buffalo, NY USA

**Keywords:** HIV, Research, Capacity building, Clinical pharmacology, Collaboration, Resource-limited

## Abstract

While important advances have been made in the prevention and treatment of Human Immunodeficiency Virus (HIV) infection, limited expertise and resource constraints to effectively manage rollout of HIV programs often contribute to poor treatment outcomes in Sub-Saharan Africa. In 1998, the University of Zimbabwe (UZ) and the University at Buffalo, State University of New York (UB), developed a collaborative clinical pharmacology capacity building program in Zimbabwe to train the next generation of HIV researchers and support rollout of the national HIV program. The collaboration was funded by research and training grants that were competitively acquired through United States of America government funding mechanisms, between 1998 and 2016. Thirty-eight research fellows were trained and a specialty clinical pharmacology laboratory was established during this period. Knowledge and skills transfer were achieved through faculty and student exchange visits. Scientific dissemination output included sixty-two scholarly publications that influenced three national policies and provided development of guidelines for strategic leadership for an HIV infection—patient adherence support group. The clinical pharmacology capacity building program trained fellows that were subsequently incorporated into the national technical working group at the Ministry of Health and Child Care, who are responsible for optimizing HIV treatment guidelines in Zimbabwe. Despite serious economic challenges, consistent collaboration between UZ and UB strengthened UZ faculty scholarly capacity, retention of HIV clinical research workforce was achieved, and the program made additional contributions toward optimization of antiretroviral therapy in Zimbabwe.

## Background

The use of potent combination antiretroviral therapy (cART) has led to successful treatment of HIV infection in most high-income countries [1]. However, treatment outcomes are often poor in sub-Saharan African countries for a variety of reasons, complicating the ability of individual countries to achieve the United Nations 95-95-95 target. The factors that contribute to these variable outcomes are diverse, but they indicate the need for building a team of researchers who are able to investigate the key clinical and implementation research questions related to optimizing HIV-1 prevention and treatment in Zimbabwe and other African countries. Challenges with financing of programs, poor health systems, suboptimal patient adherence and shortage of healthcare workers have been cited in many low to middle income countries (LMIC) [2]. Ideally, governments should commit to funding national antiretroviral programs in order to strengthen health systems, develop infrastructure and retain healthcare workers. Other capacity development projects modelled around north–south collaborations such as the African/Asian Regional Capacity Development (ARCADE) have also been useful in strengthening health systems in developing countries [3].

Retention of healthcare workers, particularly professionals with a specialist understanding of HIV and ART management is important to facilitate evidence-based optimization of ART [4]. Broadening the number of individuals who contribute to HIV care may be of value, including alternative primary care models that identify additional health providers (e.g., physician assistants, nurse practitioners, clinical pharmacists). Innovative models that integrate social and behavioural health aspects of HIV infection management have been developed. These maximize the use of a team approach to address the challenges that arise in the management of complex patients and encourage health provider led patient support groups.

In Zimbabwe, shortage of healthcare professionals has hampered the national response to the HIV epidemic, among other factors like inadequate access to health care, severe foreign currency shortages, hyperinflation, and limited industrial capacity [5–7]. The initial shortage of healthcare professionals was fuelled by brain drain as qualified personnel left the country to practice in more viable economies [8]. This in turn reduced the capacity for academic and healthcare institutions to train healthcare professionals [9, 10]. In this regard, the University of Zimbabwe (UZ) and the University at Buffalo State University of New York (UB), developed a collaborative HIV Clinical Pharmacology capacity building program whose aim was to train the next generation of HIV researchers, initially to support rollout of the national HIV program followed later by clinical and translational pharmacology research. It was conceptualised that by engaging multi-disciplinary researchers and various approaches such as fellowship programs, outcomes such as mentors for further translational training could be realised. The program would leverage on building capacity in HIV research whilst strengthening positive HIV treatment outcomes in the process. Training programs comprised of multidisciplinary fellows who would through the course of training have a strong thrust towards patient monitoring and retention. As far as can be ascertained, this is the only program that incorporated continued engagement with patients and research participants to ensure that patients who are ultimate beneficiaries of the research process would be active contributors to the research process.

This review article describes the implementation process, major achievements, challenges and the future direction of the collaboration.

## Description and implementation of the collaboration

### Description of the collaboration

The collaborative relationship between UZ and UB started in 1998, following the recommendation by the International AIDS Society (IAS) in 1997 to roll out cART. cART was identified to be the optimal treatment modality for people living with HIV even though the feasibility of providing cART in developing countries was fraught with logistical doubts [9]. One component of this collaboration was initiated to respond to the demand for cART rollout in Zimbabwe. The collaboration was subsequently formalized and managed through a memorandum of understanding (MOU) signed between UZ and UB in 2002. The MOU between UZ and UB was renewed three times between 2002 and 2017.

Based on the combined experiences and knowledge of international collaborations, the ‘bridge-building’ framework was developed (Table 1). The framework asserts the importance of building a bridge to facilitate the transfer of technological and human skills resources between highly resourced and lowly resource setting. The ‘bridge-building’ is based upon the input-process-outcomes framework. This framework was used as the analytical lens to explore the collaborative capacity building and knowledge transfer program.

**Table 1:** Bridge Building framework for the UZ and UB collaboration between 1998 and 2018

Inputs	Processes	Outcomes
**Documents** Memorandum of understanding Individual work plans **Human resources** Faculty staff Laboratory staff Computer technologists **Material resources** Laboratory equipment Laboratory reagents	Application for funding Preparation of training materials Website development HIV Research fellowship training Laboratory methods development/validation Recruitment of graduate students Formalized mentoring	Graduate students trained Joint research projects undertaken Funded projects Joint research publications Conferences and workshops attended Facilities Opportunities for higher education and scholarly internships

UB: University at Buffalo, State University of New York, New York, United States; UZ: University of Zimbabwe, Harare, Zimbabwe

### Implementation of the collaboration

**Fig. 1:**
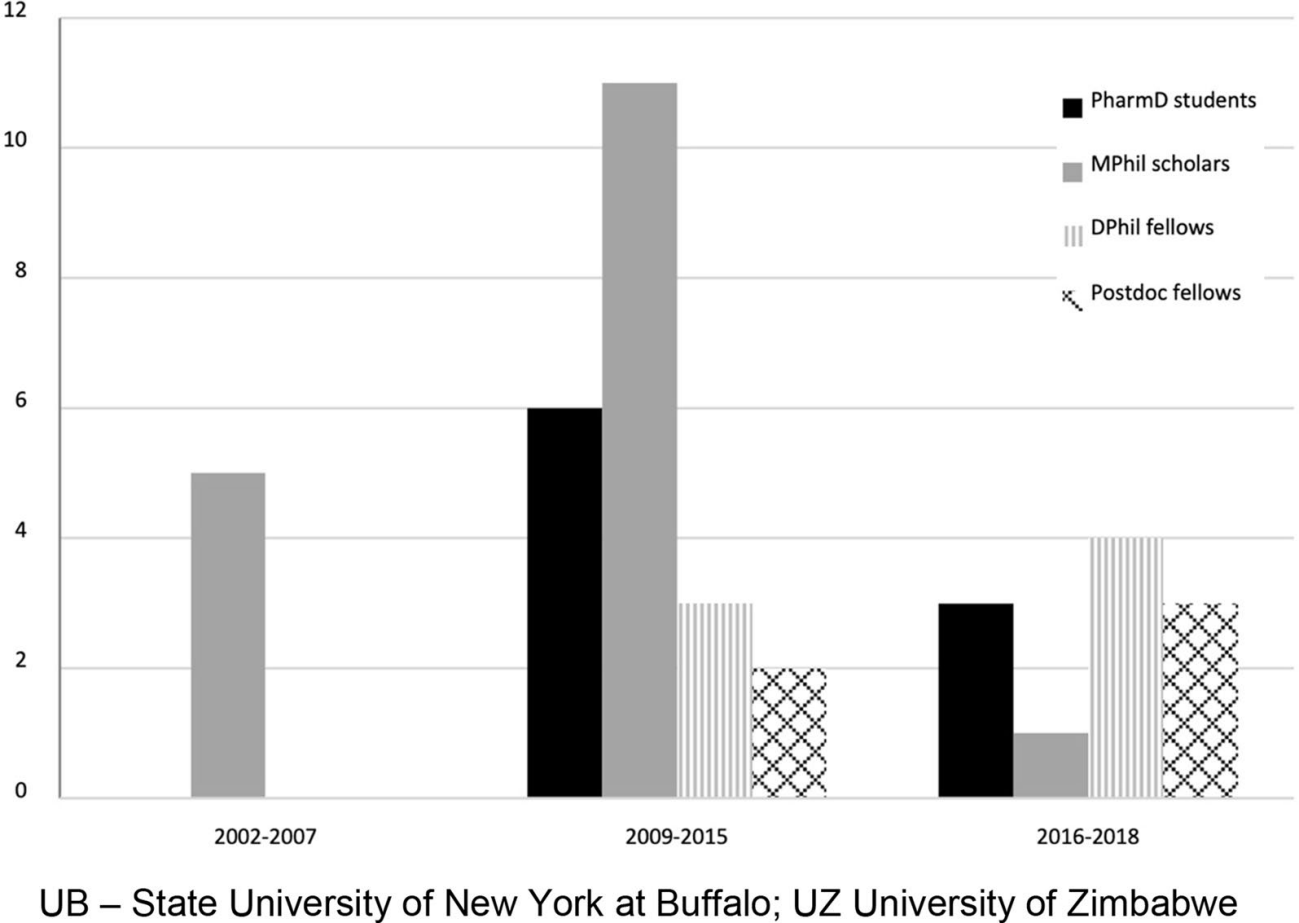
Number of student exchanges between UB and UZ from 1998 to 2018. UB: State University of New York at Buffalo; UZ: University of Zimbabwe

Faculty and students from both the UZ and UB took part in exchange visits (Fig. 1). Students studying at masters, predoctoral and postdoctoral levels received instructions and assignments from both institutions, based on assessment of self-defined learning goals, educational interests, and relevant experience. The UZ Department of Pharmacy and Pharmaceutical Sciences was represented by a designated professor who alternated between Zimbabwe and the USA to teach and share knowledge as needed. In addition to exchange visits, web based seminars between UZ and UB faculty and scholars were conducted via monthly virtual lecture hall presentations.

## Evaluation of successes of the collaboration

The capacity building program was evaluated based on the quantity of new faculty positions, and scientific dissemination was based on the number of abstracts and publications.

### Strengthening UZ faculty scholarly capacity and retention of human resources

**Fig. 2:**
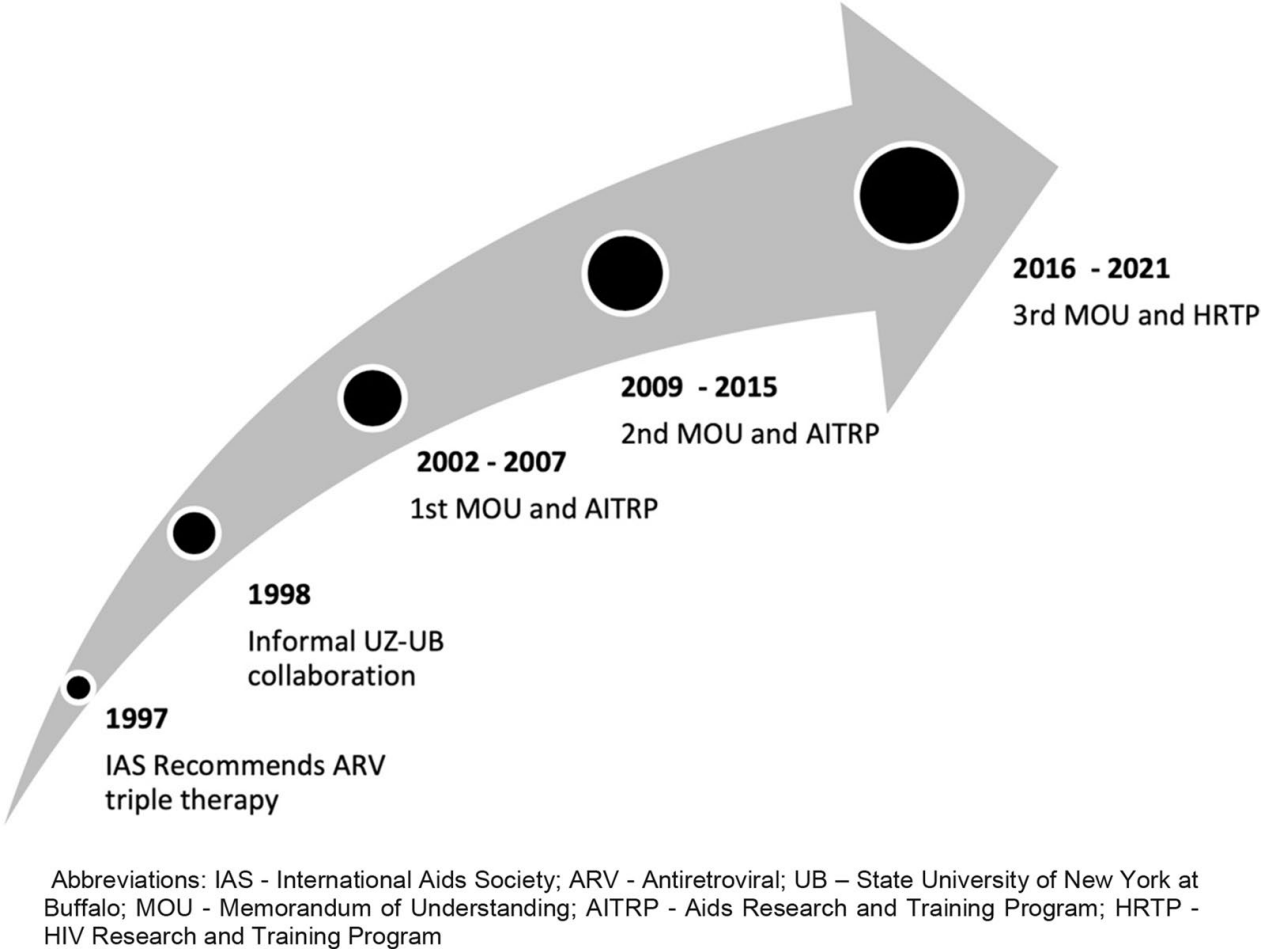
Timing of HIV Research and Training grants awarded between 1998 and 2018. IAS: International Aids Society; ARV: Antiretroviral; UB: State University of New York at Buffalo; MOU: Memorandum of Understanding; AITRP: Aids Research and Training Program; HRTP: HIV Research and Training Program

Leveraging on the MOU, the two institutions successfully competed for three NIH Fogarty International Centre HIV research and training grants (Fig. 2). The first was a two-year supplement request that was linked to the University of California, Berkeley AIDS International Training and Research Program (AITRP) in 2004. This supplement supported two initial scholars and subsequently a five-year AITRP funding was awarded to the UB and UZ to support more scholars. In a parallel AITRP award, three junior faculty members from UZ received masters level training in HIV pharmacology and public health at University of California, Berkeley and University of California, San Francisco.

**Fig. 3:**
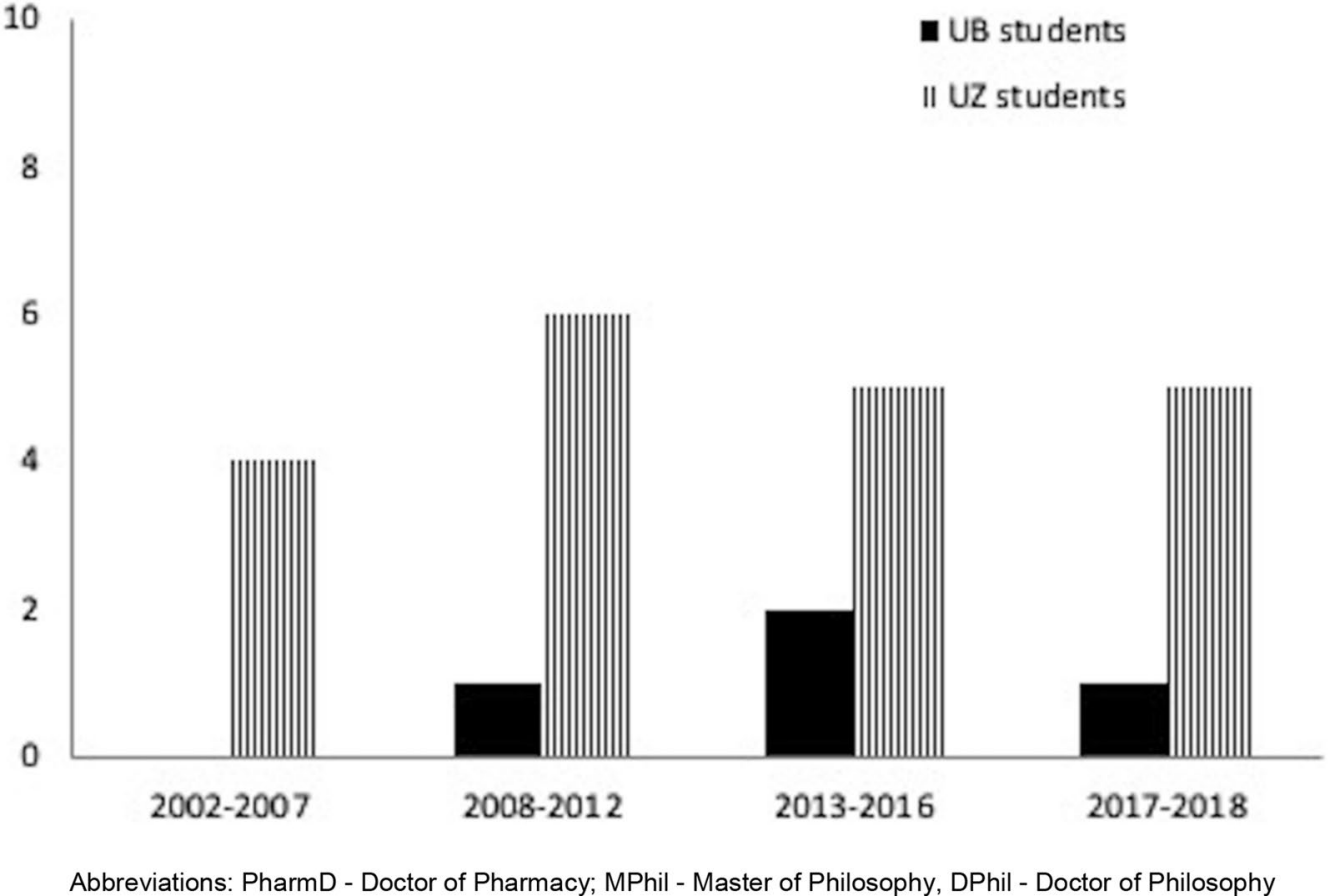
Number Graduate Fellows trained between UB and UZ from 1998–2018 by fellowship type. PharmD: Doctor of Pharmacy; MPhil: Master of Philosophy, DPhil: Doctor of Philosophy

In 2009, the UB-UZ collaborative effort renewed its MOU and was awarded a Fogarty AIDS International Training and Research Program (AITRP) grant to implement a pre-doctoral and post-doctoral training initiative with an emphasis on HIV Clinical Pharmacology between UB and UZ. During this period, two doctoral and six Masters of Philosophy students completed their studies. Further, five post-doctoral students and six PharmD students from UB were mentored in Global HIV at UZ (Fig. 3).

The current HIV Research and Training Program (HRTP) grant was awarded in April 2016. Its main thrust is the development and advancement of HIV Clinical Pharmacology training at the pre-doctoral and post-doctoral levels. Four post-doctoral scholars are currently enrolled from behavioural sciences, nanomedicine, paediatrics, genomics, and oncology with the intention of culturing the prospects of a centre for integrated global biomedical sciences. Studies in nanomedicine have been conducted by a single fellow whose focus was on combination therapies for antiretroviral and anti-tuberculosis formulations. The oncology fellow is assessing outcomes in HIV-1 infected patients with advanced cervical cancer undergoing chemoradiation. The behavioural health fellow’s research focuses on the contribution of mental, neurological conditions and psychosocial factors to treatment outcomes and deaths in HIV-infected adolescents. In addition, three scholars, psychiatrists by profession have recently been accepted to the HIV-Alzheimer’s Disease program and will complete mentored research training at UB Alzheimer’s Disease and Memory Disorders Center and focus on HIV related dementias.

The opportunity for UZ scholars to travel abroad and observe faculty in high income countries increased interest in an academic career. Scholars were attached to activities which included rotation through an international specialty laboratory, attachment to Roswell Park Cancer Institute, rotation through immunodeficiency services and translational research training. Four of the scholars joined the School of Pharmacy following research training at UB thus improving staff complement. In addition, progressive levels of training tailored to each fellow’s career development vision were offered, leading to greater career satisfaction and retention of qualified staff with research expertise in the School.

Of note, the program removed barriers to the introduction of nanotechnology research in Zimbabwe. Several successful nanoparticle fabrication projects were conducted, resulting in three publications in peer reviewed journals [11–13]. Since the inception of the program, there have been twenty publications, twenty-one poster presentations, and two special journal publications (Table 2).

**Table 2:** Scholarly outputs by UB-UZ fellows between 1998 and 2018

Output type	Number
Peer reviewed manuscripts	20
Oral abstracts	21
Posters	21
Funded grants	4
AITRP- led international workshops	5
AITRP-led TAC seminars at UB	3
NIH ACTG research meetings attended	4
Undergraduate honours projects mentored	39

UB: the University at Buffalo, State University of New York, New York, United States; UZ University of Zimbabwe, Harare, Zimbabwe; AITRP: Aids International Training Program, HRTP: HIV Research Training Program; TAC: Training Advisory Committee; NIH ACTG: National Institutes of Health Aids Clinical Trials Group

### Technology and skills transfer to support research and practice

In addition to training, the two UB-UZ training programs supported research on assessment of antiretroviral pharmacokinetics to inform optimization of cART therapy in Zimbabwe. In this regard, biomedical scientists from UB led the establishment of an international pharmacology speciality laboratory at UZ (IPSL) [14]. UB on behalf of UZ, solicited equipment from private manufacturers and donors in the US. In 2007, Waters Corporation (Milford, MA) donated a High-Performance Liquid Chromatography instrument. Another donation of an Acquity TQD Liquid chromatograph mass spectrometer was made in 2018 by Waters Corporation to expand the range of assays available in the pharmacology laboratory.

During the laboratory’s developmental period (2009–2012), AITRP scholars were trained in bioanalytical method development, validation and pharmacology aspects applicable in protocol design. Training focused on the development of chromatographic methods, laboratory quality management systems (QMS) and Clinical Pharmacology research and most was done at the Translational Pharmacology Research Core (TPRC) at UB, Buffalo, New York, United States. Additional technical training was done in-house through the NIH Division of AIDS, Clinical Pharmacology Quality Assurance (CPQA) programme after the laboratory was named to participate in this quality assurance programme in 2010 [15]. Consequently, the laboratory implemented required operational systems and methods meeting the CPQA bioanalytical method validation requirements to provide drug assay services for ACTG research protocols.

A milestone was realized in 2014 when the NIH AIDS Clinical Trials Group (ACTG) transitioned the UZ pharmacology laboratory from a developmental designation to a fully supported UZ IPSL for network research. The ACTG supports the largest network of expert clinical and translational investigators and therapeutic clinical trials units and has sites in LMIC countries including Zimbabwe.

### Strengthening the HIV clinical pharmacology evidence base for practice and policy

Publications resulting from the post-doctoral fellowship research impacted both policy and practice related to HIV management; in particular, the relevance of concomitant use of herbal medicine despite widespread availability of cART in Zimbabwe. Part of the work was instrumental in the establishment of frameworks for evaluation and implementation of herbal trial protocols and approval of herbal products for sale to the public [16, 17]. Chromatographic assays for antiretroviral drug quantitation were validated in the context of concomitant herbal medicine use. This contributed to capacity building for therapeutic drug monitoring of cART in a high herbal medicine use setting which will be valuable for both practice and research [18]. In addition, other publications highlighted potential and actual adverse drug events which may lead to poor adherence and the need for pharmacovigilance programs to expand to include herbal medicines [19, 20].

In Zimbabwe, the majority of PLHIV receive care and treatment through the national roll-out program that is run by the Ministry of Health and Child Care. Guidance for the treatment of patients is inscribed in the “Guidelines for ART for the prevention and treatment of HIV in Zimbabwe” [21]. Two fellows who graduated from the training program were invited to be part of the adaptation subcommittee for the guidelines. These fellows were invited on the basis of their specialty training/knowledge in HIV pharmacotherapy and their expertise were vital in HIV treatment optimization for the country.

## Lessons learnt from challenges

### Local partnerships support international collaboration

Recruitment and retention of participants for scholars’ research was a challenge initially. Collaboration with a community-based patient support group was important in building interest in the Fogarty International Center scholars’ research projects. The Perseverance Adherence Respect and Integrity (PARI) support group played a key role in the sensitization, recruitment and retention of study participants for research projects. The Parirenyatwa Hospital outpatient HIV clinic provided space for the PARI support group to meet. Fellows from both UZ and UB gave back to the support group by providing up-to-date health education information and responding to members concerns regarding ART in general. The drug information service resulted in improved medication adherence and patient retention in care [22, 23].

Laboratory facilities were initially provided by UZ to establish the International Pharmacology Speciality Laboratory. However, with the addition of two High Pressure Liquid Chromatography systems, the space became inadequate. An agreement was made in 2012 with the national drug regulatory authority, Medicines Control Authority of Zimbabwe (MCAZ), to house the laboratory at their premises. Cognisant of its limitations, the Government of Zimbabwe has in recent years advocated for public private partnership paradigm shift. Collaborations between private and public institutions to address some of these challenges have yielded positive results [24].

Initially, fellows movement had been planned in one direction, i.e., south to north. As the global health agenda developed, the HRTP appealed to UB students interested in contributing to LMIC global health programs. The local practice context and networks established by HRTP were valuable in supporting training of international Global Health HIV scholars.

### Retention of fellows, mentors and support staff

Despite initial efforts to establish strategies for fellow and faculty retention, the country’s unstable economic environment threatened continuity of the program. Both the local private sector or other countries offered better financial security for fellows, mentors and support staff. In order to avoid reversing gains, subsequent grants offered competitive stipends, honoraria and salary thereby ensuring continuity. This proved to be a retention strategy not only for UZ but also for the national HIV program and the MCAZ where several of the fellows also held important posts as expert support.

While this collaboration has been critical in establishing a framework for building capacity in treatment, training and research programmes, it has also led to the retention and development of a critical mass of technically competent researchers who are capable of advancing the research agenda at international level. This was an important lesson learnt when implementing the research and training program through collaborative efforts. Fellows and scholars in the program were able to access resources, mentorship and facilities from high income countries. Consequently this led to a significant decrease in the exodus of scientists to other countries in search of better resources.

Through collaborative agreements, low resource settings are in a position to gain through technology and instrumentation transfer. Whilst the collaboration was originally set up as a training and research program, technology transfer ensued due to the existence of the framework. Finally and more importantly, mentorship in the program has led to the development of a mentorship culture within the local setting. Fellows who have gone through the program become mentors as well to other scientists in training. Consequently, the benefits of the collaboration have yielded positive results beyond those trained in the program.

## Conclusions and future plans

Despite the economic challenges, consistent collaboration between the UZ and UB contributed to strengthening UZ faculty scholarly capacity, retention of HIV workforce in Zimbabwe and building the HIV Clinical Pharmacology evidence base. Through the training program, fellows have occupied strategic positions providing technical expertise and influencing policy resulting in optimization of HIV treatment. Capacity building programs in Zimbabwe that use HIV research training to establish a foundation have now led to additional focus areas in cancer and neuropsychology that also contribute to the team science approach. Since its inception, the training program has been consistent, persistent and continues to scale upwards. In the next cycle, the program aims to generate Centres of Excellence in Pharmaceutical Innovation on a regional scale to drive advanced pharmaceutical inventions and innovation across Africa and the world at large.

## Data Availability

All data and materials related to this manuscript are available for review by request to the corresponding author.

## Abbreviations

ARCADE: African/Asian Regional Capacity Development
ACTG: AIDS Clinical Trials Group
AITRP: AIDS International Training and Research Program
CPQA: Clinical Pharmacology Quality Assurance
cART: Combination ART
HRTP: HIV Research and Training Program
HIV: Human Immunodeficiency Virus
IAS: International AIDS Society
IPSL: International pharmacology speciality laboratory
IPERI: International Pharmacotherapy Education and Research Initiative
LMIC: Low to Middle Income Countries
MCAZ: Medicines Control Authority of Zimbabwe
MOU: Memorandum of understanding
PARI: Perseverance Adherence Respect and Integrity
QMS: Quality management systems
TPRC: Translational Pharmacology Research Core
UB: University at Buffalo, State University of New York
UZ: University of Zimbabwe
